# Evaluation of the mannan antigen assay in neonates with or without *Candida albicans* colonization

**DOI:** 10.1093/mmy/myad138

**Published:** 2024-01-02

**Authors:** Vasiliki Bourika, Tania Siahanidou, Kalliopi Theodoridou, Athanasios Tsakris, Georgia Vrioni, Athanasios Michos

**Affiliations:** Neonatal Unit, First Department of Pediatrics, National and Kapodistrian University of Athens, Athens, Greece; Neonatal Unit, First Department of Pediatrics, National and Kapodistrian University of Athens, Athens, Greece; Department of Microbiology, Medical School, National and Kapodistrian University of Athens, Athens, Greece; Department of Microbiology, Medical School, National and Kapodistrian University of Athens, Athens, Greece; Department of Microbiology, Medical School, National and Kapodistrian University of Athens, Athens, Greece; Division of Infectious Diseases, First Department of Pediatrics, National and Kapodistrian University of Athens, Athens, Greece

**Keywords:** mannan, *Candida*, carriage, infant, neonate

## Abstract

Mannan antigen (MA) in neonates as a marker of invasive candidemia is not well studied, although 4% of all neonatal intensive care unit admissions are attributed to *Candida* spp. infections. The aim of this case-control study was to evaluate the performance of MA (Platelia™ Candida AgPluskit, Bio-Rad) in neonates who had rectal *Candida* colonization or in non-colonized controls. We cultured 340 rectal swabs of neonates and MA was negative in 24/25 *C. albicans* colonized (96% specificity) and in 30/30 non-colonized neonates (100% specificity). The results indicate a high specificity of the assay, which could be useful in neonates with possible candidemia.

## Introduction

It is estimated that almost 4% of all NICU (Neonatal Intensive Care Unit) admissions are attributed to *Candida* spp. infections.^1,2^*Candida albicans* is the leading cause of neonatal candidiasis,^1,3^ while *Candida parapsilosis* is the most common non-albicans isolate in VLBW (very low birth weight) infants. Invasive candidiasis (IC) in neonates is associated with considerably increased mortality,^1^ which is further worsened when the pathogen is isolated from multiple body sites.^4^

Blood cultures^3^ remain the gold standard for the diagnosis of invasive fungal infection (IFI), even though they require time and may exhibit less sensitivity under certain circumstances.^4,5^

The use of mannan antigen (MA) assay in neonates is not well studied. The aim of our study was to evaluate the performance and the specificity of MA in hospitalized neonates (age < 28 days).

## Materials and methods

This was a prospective, case-control study of hospitalized neonates in the Special Care Neonatal Unit (Level II) of the 1st Pediatric Department, National and Kapodistrian University of Athens, ‘Aghia Sophia’ Children’s Hospital. Late pre-term (>34 weeks) or term infants are hospitalized in this unit, and the incidence of invasive fungal infections is low. For this reason, the primary objective of the study was to evaluate the specificity of the MA assay in *Candida* colonized and non-colonized infants. Only digestive colonization through rectal cultures and not cutaneous has been taken into account.

Rectal samples for *Candida* colonization were collected on admission and blood samples were drawn for routine blood tests and blood cultures.

Blood sample for MA detection was collected during the first 3 days of hospitalization. Serum was collected and stored at −20 °C until assayed. Detection of MA was carried out using the commercially available Platelia™ Candida Ag Plus kit (Bio-Rad), according to the manufacturer’s instructions. A negative result, as defined by the manufacturer, was with concentrations <62.5 pg/mL, while samples with concentrations between 62.5 and 125 pg/mL were considered to be “intermediate” for MA, and those with concentrations that were ≥125 pg/mL were considered to be “positive” for MA. All samples were run in duplicate. Analysis of the results was carried out blind to the clinical and microbiological data.

The study protocol was approved by the ‘Aghia Sophia’ Children’s Hospital Ethics Committee (study approval number: 5878), and informed written consent was obtained from infants’ parents.

Statistical analysis was performed using SPSS version 28.0 (SPSS Inc, Chicago, IL, USA).

## Results

A total of 340 neonates were included in the study, and culture for rectal *Candida* colonization was positive in 39/340 (11.47%). In all colonized neonates only *C. albicans* was identified. Blood sample was drawn for MA in 25/39 neonates (for 14 neonates, there was no written parent consent). A total of 30 neonates, who were hospitalized at the same period, with negative result for *Candida* rectal colonization and similar distribution of age and sex, were included as controls.

The characteristics of the study population are presented in Table 1. The mean ± SD gestational age of the study population was 38.2 ± 1.4 weeks, 34 neonates (62%), had vaginal birth, 29/55 neonates were male (52.7%) and at the time of the study were 17.3 ± 7.8 days of age.

**Table 1.** Epidemiological characteristics of neonates who were positive or negative for *C. albicans* carriage.

	Total population (*N* = 55) *n* (%)	*C. albicans* colonized (*n* = 25) *n* (%)	*C. albicans* non-colonized (*n* = 30) *n* (%)	*P*-value
**Gender**				
**Female**	26 (47.3)	12 (48)	14 (46.7)	0.568
**Male**	29 (52.7)	13 (52)	16 (53.3)	
**Labor**				
**Cesarean section**	21 (38)	10 (40)	11 (36)	0.574
**Vaginal birth**	34 (62)	15 (60)	19 (64)	
**Gestational age (wks)**	38.2 ± 1.4	38.2 ± 1.5	38.3 ± 1.4	0.920
**Birthweight (gr)**	3003.9 ± 785.2	3106.3 ± 537.9	2905.6 ± 967	0.719
**Days at maternity hospital**	3.3 ± 1.4	3.5 ± 1.8	3.2 ± 0.6	0.891
**Age (days)**	17.3 ± 7.8	18.7 ± 8.6	16.0 ± 6.7	0.193
**Duration of antibiotics**				
**None**	32 (58)	9 (36)	23 (77)	0.025
**≤24 h**	13 (24)	7 (28)	6 (20)	
**24-48 h**	6 (11)	5 (20)	1 (3)	
**>48 h**	2 (3.5)	2 (8)		
**Before hospitalization**	2 (3.5)	2 (8)		

Quantitive variables are expressed as mean ± SD.

The leading causes of admission in both groups were bronchiolitis (36%), jaundice (16.4%), fever (11%), failure to thrive (9%), vomiting (7.3%), irregular breathing or cyanosis (5.5%), diarrhea (3.6%), late prematurity (3.6%), opthalmia neonatorum (3.6%), rash (2%), and heart murmur (2%).

The laboratory results are presented in Table 2. During the study period, no invasive fungal infection was detected in both the *Candida*-colonized and non-colonized neonates.

**Table 2.** Cultures (blood, urine, and CNF), full blood count, and CRP levels in neonates who were positive or negative for *C. albicans* carriage.

	Total population (*N* = 55) *n* (%)	*C. albicans* colonized (*n* = 25) *n* (%)	*C. albicans* non-colonized (*n* = 30) *n* (%)	*P*-value
**Blood culture for bacteria**				
**Positive**	1 (98.2)	1 (4)	0 (0)	0.5
**Negative**	54 (1.2)	24 (96)	30 (100)	
**Blood culture for fungi**				
**Positive**	0	0	0	
**Urine culture**				
**Positive**	6 (11)	3 (12)	3 (10)	0.25
**Negative**	49 (89)	22 (88)	27 (90)	
**CSF culture**				
**Positive**	0	0	0	
**WBC (/µL)**	12 029 ± 4266	11 728 ± 3007	12 318 ± 5248	0.984
**Neutrophils (%)**	33.5 ± 13.3	34 ± 13.5	33 ± 13.0	0.897
**Lymphocytes (%)**	47.5 ± 13.0	46.5 ± 12.6	48.4 ± 13.6	0.627
**Monocytes (%)**	12.2 ± 3.7	12.8 ± 4.1	11.6 ± 3.4	0.272
**Hb (gr/dL)**	13.9 ± 2.0	13.5 ± 1.8	14.3 ± 2.2	0.167
**Hct (%)**	42.8 ± 6.5	41.4 ± 5.8	44.08 ± 7.0	0.152
**PLT (*x* 10^3^/mm^3^)**	390 ± 113.1	418.3 ± 114.1	362.7 ± 107.4	0.085
**CRP (mg/L)**	6.4 ± 18.5	6.5 ± 13.2	6.3 ± 22.7	0.562

Quantitive variables are expressed as mean ± SD.

CSF, cerebrospinal fluid; WBC, whole blood count; Hb, hemoglobin; Hct, hematocrit; PLT, platelet count; CRP, C-reactive protein.

MA was negative in all uncolonized neonates (30/30, 100% specificity) and in 24/25 *C. albicans* colonized neonates (96% specificity). Only one *C. albicans*-colonized neonate, was detected positive with the MA assay, who was admitted at 13 days of postnatal age for fever and has started oral topical miconazole treatment for oral *Candida* mucositis. The neonate, who was the only one with mucocutaneous mucositis in the total population, had no signs of invasive candidiasis; cultures were negative and blood culture for IFI was also negative.

## Discussion

The present study aimed to evaluate the specificity and performance of MA assay in neonates hospitalized in a Level II neonatal unit. For this reason, we compared the specificity of the MA assay in neonates with or without rectal *C. albicans* colonization. According to our results, none of the non-colonized infants had positive MA, whereas only one from the colonized group had positive result. However, this neonate had oral *Candida* mucositis, which could possibly facilitate the presence of the antigen in the blood. During the study period there was not any IFI.

In the present study, laboratory results did not differ between colonized and non-colonized neonates. Furthermore, full blood count, including platelets, and CRP did not differ between positive and negative carriers. The role of laboratory findings and/or biomarkers in the accurate diagnosis of IC has been evaluated in a clinical study of Guo et al.,^6^ in which white blood cell count, platelet count, hs-CRP levels, PCT, and β-D-glucan (BDG) were compared between 30 neonates with IC, 25 neonates with bacterial infection, and 25 neonates considered as controls. The study reported that, WBCs and PCT levels did not differ between neonates with IC compared to controls. On the contrary, CRP levels were statistically significant higher and platelet count was statistically significant lower in neonates with IC compared to controls. BDG was the only biomarker that was found statistically higher in neonates with IC compared to neonates with bacterial infection or controls.^6^ BDG, also may aid to the diagnosis of IC;^6^ however, it should be noticed that is not specific and can also be detected in patients with bacteremia either from gram-positive or gram-negative pathogens.^5^

The performance of MA has been previously studied in adults and neonates. The third European Conference on Infections in Leukemia meeting conducted a systematic literature review,^7^ including 14 studies of adult patients with hemato-oncology diseases and invasive candidiasis, and tried to evaluate the sensitivity and specificity of MA and mannan antibody. According to their results, there is significant heterogeneity among studies; however, the combined test of MA and mannan antibody performed better with 83% sensitivity and 86% specificity.

Regarding neonates, according to the study by Oliveri et al.^8^ MA was considered positive in at least two samples with levels >0.5 ng/mL. The MA was negative only in 1 patient out of 12, who was diagnosed with invasive candidiasis by *C. parapsilosis*. It is remarkable that in 8/12 neonates, the antigen was positive before the blood culture. However, 3 neonates out of 58 without candidiasis had false-positive MA. According to the above results, the sensitivity of the assay was 94.4% and the specificity was 94.2%. Furthermore, in a multi-center survey in NICU in southern Italy in 2010, MA was positive in five out of seven neonates with IFI.^9^ However, there is no recent study evaluating possible MA presence in the serum of neonates with rectal colonization.

Limitations of the present study include that in the population, there were not many early preterm or VLBW neonates, which may explain that we did not detect any IFI episodes, even in the *C. albicans* colonized neonates. For this reason, we could not estimate the sensitivity of the assay for the diagnosis of IFI. However, the importance of the study is that we found a very high specificity in both colonized and non-colonized babies.

Further investigation is needed in high-risk neonates, that have higher propability of IFI, to estimate the sensitivity of the MA assay.

## Conclusions

MA was found to have high specificity in neonates, regardless of rectal *Candida* colonization status. That is important for further evaluation of the assay for the detection of invasive fungal infection in neonatal units and the timely administration of antifungals.
